# Eukaryotic translation initiation factors as promising targets in cancer therapy

**DOI:** 10.1186/s12964-020-00607-9

**Published:** 2020-11-04

**Authors:** Peiqi Hao, Jiaojiao Yu, Richard Ward, Yin Liu, Qiao Hao, Su An, Tianrui Xu

**Affiliations:** 1 Faculty of Environmental Science and Engineering, Kunming University of Science and Technology, 727 Jingming South Road, Kunming, 650500 China; 2 Faculty of Life Science and Technology, Kunming University of Science and Technology, Kunming, 650500 China; 3 Molecular Pharmacology Group, Institute of Molecular, Cell and Systems Biology, College of Medical, Veterinary and Life Sciences, University of Glasgow, Glasgow, G12 8QQ Scotland, UK

**Keywords:** eIF, mRNA translation, Cancer, MAPK, PI3K/Akt, mTOR

## Abstract

The regulation of the translation of messenger RNA (mRNA) in eukaryotic cells is critical for gene expression, and occurs principally at the initiation phase which is mainly regulated by eukaryotic initiation factors (eIFs). eIFs are fundamental for the translation of mRNA and as such act as the primary targets of several signaling pathways to regulate gene expression. Mis-regulated mRNA expression is a common feature of tumorigenesis and the abnormal activity of eIF complexes triggered by upstream signaling pathways is detected in many tumors, leading to the selective translation of mRNA encoding proteins involved in tumorigenesis, metastasis, or resistance to anti-cancer drugs, and making eIFs a promising therapeutic target for various types of cancers. Here, we briefly outline our current understanding of the biology of eIFs, mainly focusing on the effects of several signaling pathways upon their functions and discuss their contributions to the initiation and progression of tumor growth. An overview of the progress in developing agents targeting the components of translation machinery for cancer treatment is also provided.

**Video abstract**

**Supplementary Information:**

The online version contains supplementary material available at 10.1186/s12964-020-00607-9.

## Background

The regulation of gene expression in eukaryotes can occur at different stages including gene transcription and mRNA translation. In comparison with transcriptional control, translational regulation of pre-existing mRNAs provides more direct, rapid and sensitive changes in intracellular levels of the encoded proteins and thus cellular adaptation during physiological and pathological conditions by rapidly reprogramming the proteome expression without the requirement for changes in RNA synthesis. Eukaryotic mRNA translation is a very complicated process that consists of four major phases:initiation, elongation, termination and ribosome recycling, while the regulation takes place mainly at the initiation stage which is the rate-limiting step of protein synthesis among the four steps of translation [1]. The major regulators in the initiation stage are the eukaryotic translation initiation factors (eIFs). In fact, eukaryotes utilize many more initiation factors than do prokaryotes, reflecting the greater biological complexity of eukaryotic translation. First, in eukaryotes, the 43S ribosomal pre-initiation complex (PIC) which is composed of the 40S ribosomal subunit, the eIF2-GTP-initiating methionyl tRNA (Met-tRNAi) ternary complex and many other eIFs including eIF1, eIF1A, eIF3 and eIF5, is recruited to the 5’ terminus of the mRNAs, and is thought to scan the 5’ untranslated region (5’UTR), then the PIC moves towards the start codon, where the 60S ribosomal subunit joins the complex resulting the formation of the 80S initiation complex. Then the 80S complex is prepared to recruit the correct aminoacyl-tRNA into the A (aminoacyl) site, promoting synthesis of the first peptide bond and shifting initiation towards the elongation step [2].

During this process, eIFs can assist with the stabilization of the functional 80S initiation complex at the start codon and also act as regulatory targets for translation initiation. Translation and translational regulation are recognized as a key node in inducing adaptive stress responses to conquer various stress conditions imposed on cancer cells by the tumor microenvironment, immunological surveillance, their continuous proliferation and cytotoxicity of antitumor drugs. It is well known that mis-regulation of many eIFs most frequently contributes to tumorigenic transformation, cancer development and progression, and is of the utmost interest when targeting cancer [2]. Studies in the past two decades have indicated that a group of initiation factors such as eIF4, eIF3, eIF2 and eIF5 are implicated in various types of cancer [3–6]. Additionally, eIFs have been shown to contribute to the hallmarks of cancer, including sustained proliferative signaling, uncontrolled growth, angiogenesis, invasion, metastasis, resistance to apoptosis and replicative immortality [7]. The expression level, availability and activity eIFs, which are usually regulated by several key signaling pathways, such as phosphatidylinositol 3-kinase (PI3K)/AKT, mitogen-activated protein kinase (MAPK), and mammalian target of rapamycin (mTOR) pathways, have significant effects on translation initiation. As such, mRNA translation is strictly regulated by signal pathways which can sense and respond to both environmental and intracellular stimuli. In this review, we describe our current knowledge about the basic functions of eIFs in translation initiation, in particular, discussing the crucial roles of eIF mis-regulation and the multiple regulatory pathways of eIF functions in tumorigenesis and tumor progression. The prognostic value of eIF perturbation in cancers and the possibility of eIFs serving as potential targets for the treatment of cancers are also explored.

## Overview the role of eIFs in translation initiation

**Fig. 1:**
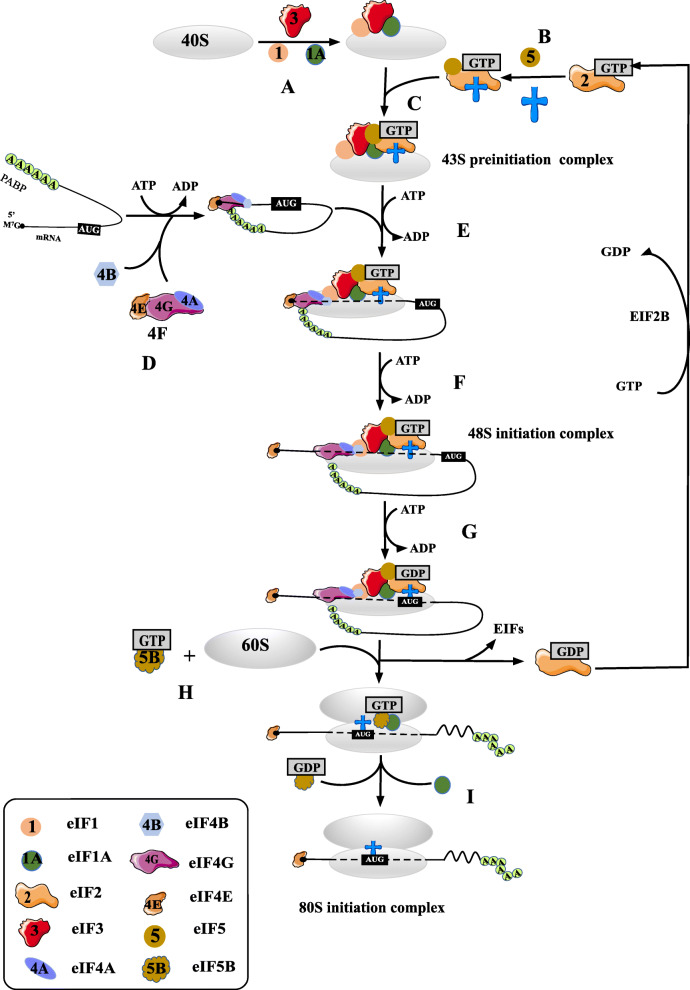
Schematic representation of the pathway of eukaryotic translation initiation. The whole process of eukaryotic translation initiation can be divided into nine stages: **a** the recycling of separated ribosomal subunits and eIFs which are generated from the previous mRNA translations. **b** the formation of eIF2-GTP-Met-tRNAi^Met^ ternary complex. **c** the formation of 43S PIC which is composed of eIF2-GTP-Met-tRNAi^Met^ ternary complex, 40S ribosomal subunits, eIF1, eIF1A, eIF3 and eIF5. **d** the activation of mRNA by eIF4F complex with the assistance of eIF4B, eIF3 and PABP. **e** the attachment of 43S PIC to mRNA. **f** the scanning of mRNA 5’UTR in a 5’-3’ direction by 43S PIC. **g** the recognition of start codon and the formation of 48S initiation complex. **h** the jointing of 60S ribosomal subunits to the 48S complex with the assistance of eIF5B-GTP and eIF1A, and the concomitant displacement of eIF2-GDP and other factors including eIF1, eIF3, eIF4B, eIF4F and eIF5. **i** hydrolysis of eIF5B-bound GTP and release of eIF1A and eIF5B-GDP from the 80S ribosome, mRNA translation enters the elongation stage

Translation initiation in eukaryotes is, as described previously, the most highly regulated phase in the translation of most mRNAs, leading to the assembly of an elongation-competent 80S ribosome through the join of the large (60S) ribosomal subunits to the small (40S) ribosomal subunits with the Met-tRNAi positioned around the start codon (Fig. 1). Initiation begins with the formation of the eIF2-GTP-Met-tRNAi ternary complex, which then assembles with the 40S ribosomal subunit, eIF1, eIF1A, eIF3 and probably eIF5 to form a 43S PIC [2] (Fig. 1).

Then the PIC is recruited to the 5’ end of mRNA, labeled with by an inverted m7GpppN cap [8]. Prior to the attachment of PIC to this mRNA region, mRNA needs to be unwound or activated by the eIF4F complex consisting of the cap-binding protein (eIF4E), RNA helicase (eIF4A) and eIF4G with the assistance of eIF4B, eIF3 and PABP. The association of regulating factor eIF4B with eIF4A significantly enhances its helicase activity [2]. Once attached to the mRNA, the 43S PIC is considered to scan on the 5’-untranslated region (5’UTR) in the 5’ to 3’ direction until the start codon is recognized and the 48S initiation complex formed. Once PIC recognizes the start codon, eIF1 is released, permitting the hydrolysis of eIF2-bound GTP and Pi release mediated by eIF5 [9] (Fig. 1).

These processes prompt the transition of PIC from an ‘open’ conformation to a ‘closed’ conformation, the latter stabilizing the interaction of PIC with mRNA. Then eIF5B-GTP collaborates with the eIF1A to assist in the formation of 80S ribosomal initiation complex through the recruitment of the 60S subunit to the 48S initiation complex, a process which is accompanied by the release of eIF1, eIF2-GDP, eIF3, eIF4B, eIF4F and eIF5. Subsequent hydrolysis of GTP by eIF5B and the displacement of eIF5B-GDP and eIF1A from assembled 80S ribosome make the complex ready to enter the elongation phase of protein synthesis [2] (Fig. 1).

Signaling pathways promoting tumorigenesis possess growth factor signaling characteristics which strongly stimulate the activation of receptor tyrosine kinases (RTKs), MAPK and PI3K/AKT signaling pathways [2]. These pathways play a significant role in the regulation of eIF functions, indeed their mis-regulation usually causes aberrant translation, finally leading to tumorigenesis. To date, the roles of MAPK and PI3K/AKT signaling pathways in the regulation of eIFs are the best-characterized regulatory mechanism. Interestingly, both MAPK and PI3K/AKT pathways employ the mammalian target of rapamycin (mTOR) to regulate the functions of eIFs. Therefore, mTOR plays a leading role in the regulation of eIF functions and protein synthesis, and is recognized as the master regulator of protein synthesis and cell proliferation [10].

## mTOR

**Fig. 2:**
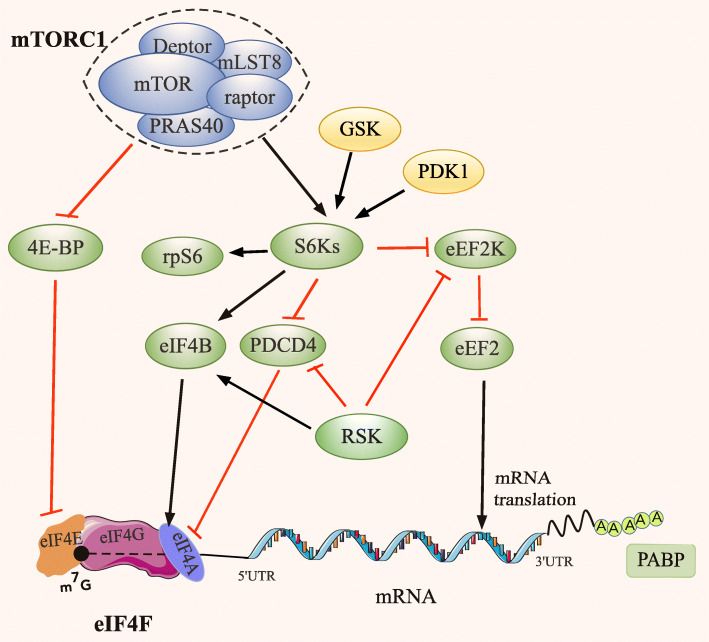
The major substrates of mTORC1 and their signaling to the translational machinery. 4E-BPs and S6Ks are the two major mediators of the effects of mTORC1 on mRNA translation. In non-phosphorylated states, 4E-BPs block the assembly of the eIF4F complex by competing with eIF4G for binding to eIF4E. When phosphorylated by mTORC1, the hyper-phosphorylation of 4E-BPs facilitates their dissociation from eIF4E, allowing the interaction of eIF4E and eIF4G and the assembly of eIF4F complex. In addition to 4E-BPs, S6Ks also mediate the effects of mTORC1 on mRNA translation. The major S6Ks substrates involved in the regulation of translation are rpS6, eIF4B, eEF2K and PDCD4, which are also phosphorylated by other AGC kinases including RSK and AKT. rpS6 is a component of the 40S ribosomal subunit, and eIF4B is an auxiliary factor that enhances the RNA-unwinding activity of eEF4A. The phosphorylation of rpS6 and eIF4B by AGC kinases significantly promote the translation of mRNA. PDCD4 is reported as pro-apoptotic factor and has been suggested to possess tumor suppressor properties. eEF2K functions as a negative regulator of protein synthesis via phosphorylation and inhibition of eEF2. The phosphorylation of PDCD4 and eEF2K by AGC kinases leads to PDCD4 degradation and the inhibition of eEF2K kinase activity, respectively. Black arrows and red T-bars represent stimulatory and inhibitory signals, respectively

mTOR is a conserved Ser/Thr kinase that orchestrates a broad spectrum of environmental and intracellular stimuli including growth factors, hormones and metabolic alterations to adjust growth and proliferation [11]. In mammals, mTOR is found to have two structurally and functionally distinct multiprotein complexes, named mTOR complex 1 (mTORC1) and mTOR complex 2 (mTORC2). mTORC1 is defined by its three core components: mTOR, Raptor (regulatory protein activated with mTOR) and mLST8 (mammalian lethal with Sec13 protein 8, also known as the GTPase β-subunit like protein, GβL) [12] (Fig. 2).

Raptor, a specific component of mTORC1, endows the substrate specificity of mTORC1, in part by assisting in substrate recruitment to mTORC1 through interacting with several mTORC1 substrates [12, 13]. Whereas mLST8 interacts with the catalytic kinase domain of mTORC1, and may promote the stabilization of kinase activation loop, though *mLST8* gene knockout studies indicated that it is unnecessary for the fundamental functions of mTORC1 [14].

In addition, mTORC1 also includes the other four associated proteins, PRAS40, Deptor (DEP domain-containing mTOR-interacting protein), Rags A/B/C/D and Rheb. Two of the subunits, PRAS40 and Deptor [15], are negative regulators of mTORC1. PRAS40 has a functional TOR signaling (TOS) motif that associates with Raptor and thus may interfere with the binding of other mTORC1 targets to Raptor [16]. After phosphorylation by AKT or mTORC1, PRAS40 will dissociate from mTORC1 thus removing its inhibitory effects [17]. Rag GTPases, which belong to the Ras superfamily GTP-binding proteins, recruit mTORC1 to the lysosomal surface facilitating mTORC1 activation by Rheb [18]. Like mTORC1, mTORC2 also contains mTOR, mLST8 and Deptor. Instead of Raptor, however, mTORC2 contains Rictor (rapamycin insensitive companion of mTOR), as well as the two regulatory subunits mSin1 and Protor1/2.

The different components and structures of mTORC1 and mTORC2 allow for distinct functions. For example, mTORC1 is relatively sensitive to rapamycin, while mTORC2 is relatively rapamycin resistant. In terms of protein synthesis, mTORC2 bind selectively to ribosomal membranes, where it can interact with its key substrates, the ACG kinases such as AKT, protein kinase C (PKC), and serum glucose kinase (SGK), and then promote their activity. In contrast, mTORC1 mainly associates with endosomal and lysosomal membranes, where it promotes the phosphorylation and activation of downstream substrates, 40S ribosomal protein S6 kinases (S6Ks) and 4E-binding protein 1 (4E-BP1). When considering their effects on eIFs functions, the one that is mainly involved in translational control and the regulation of eIFs functions is mTORC1, S6Ks and 4E-BP1 are the most extensively characterized and best-understood downstream substrates of mTORC1 [19].

## The functions of eIFs regulated by mTORC1

mTORC1 plays a critical role in stimulating global protein synthesis via regulating the translation of proliferation related, survival and tumor promoting mRNAs, as well as the house-keeping mRNAs. The best-understood effectors of mTORC1 in translation are 4E-BPs and S6Ks [20–22] (Fig. 2).

### 4E-BPs

Cap-dependent translation initiation start from the assembly of eIF4F complex on the 5’-mRNA cap structure which is indispensable for recruiting mRNAs to ribosomes [23]. The eIF4F complex is a heterotrimer consisting of eIF4E (a cap-binding subunit), eIF4G (a scaffold protein) and eIF4A (a RNA helicase). eIF4E serves as the cap-binding component, eIF4A acts as an ATP-dependent RNA helicase which unwinds mRNA secondary structure [24]. eIF4G is a large scaffolding protein which act as molecular bridges between eIF4E and eIF4A, and also interacts with PABP and eIF 3[23, 24]. 4E-BPs including 4E-BP-1, 4E-BP-2 and 4E-BP-3 in mammals are negative translational regulators [25]. In their non-phosphorylated states, 4E-BPs impede the eIF4F complex formation through competing with eIF4G for the same binding site on eIF4E [26].

Upon activation, mTORC1 stimulates the hierarchical phosphorylation of 4E-BPs, such as the phosphorylation of Thr37 and Thr46 in human 4E-BP1 which leads to the further phosphorylation of Ser65 and Thr70 [27, 28]. 4E-BPs are also phosphorylated by cyclin B-CDK1 (Cyclin Dependent Kinase 1). The hyper-phosphorylation of 4E-BPs facilitates their disaggregation from eIF4E, thus allowing the association between eIF4E and eIF4G, and the formation of the eIF4F complex. It has been reported that phosphorylated 4E-BP1 levels in breast, ovary and prostate tumors is closely related to malignant development and poor prognosis regardless of the difference of upstream oncogenic stimulations [29].

Although eIF4E is a general translation initiation factor essential for cap-dependent translation initiation in eukaryotes [25], it is well known that the changes of eIF4E expression level and activity affect the translation of a specific subset of eIF4E-sensitive mRNAs, but do not influence the global protein expression. It is thought that eIF4E-sensitive mRNAs which are characterized by the long, complex and highly structured 5’-UTR mainly encoding proliferation and tumor-promoting proteins such as Bcl-xL, C-myc, Cyclins and vascular endothelial growth factor (VEGF) [30]. As the least abundant and most rate-limiting eukaryotic translation initiation factor, eIF4E controls the level of eIF4F complex. Therefore, mTORC1 can enhance the translational activity of eIF4E and stimulate the translation of eIF4E-sensitive mRNAs through the phosphorylation and inactivation of 4E-BPs [30].

### S6Ks

Besides 4E-BPs, S6Ks also play an important role in mediating the effects of mTOR on mRNA translation [31, 32]. There are two variants of S6K in mammals, S6K1 (S6Kα) and S6K2 (S6Kβ) [33], and the two kinases encoded by two independent genes share a high degree of homology [34]. Additionally, both S6K1 and S6K2 exhibit two distinct isoforms (p70- and p85-S6K1, p54- and p56-S6K2), which are produced via alternative transcription start site selection. The more abundant S6K1 isoform is p70-S6K1 which is predominantly localized in cytoplasm, whereas p85-S6K1 and both S6K2s are mainly to be found in the nucleus [35]. It is noteworthy that S6K1/2 double knockout mice have small body size and suffer from perinatal lethality [36], whilst small-body size is noticed in S6K1-deficient mice, but not in S6K2-deficient mice [36]. Similarly, a single S6K isoform knockout mouse embryo fibroblasts and myoblasts exhibit abnormality in size but not propagation [37]. Thus, it is suggested that S6K1/2 have overlapping as well as non-redundant functions.

It has been observed that S6K1 plays an important role in control of oncogenic processes in estrogen receptor (ER)-positive breast cancer cells. S6k1 directly phosphorylates and activates ERα and in addition S6K1 expression is regulated by estrogen [38]. However, in small cell lung cancer, it is S6K2 but not S6k1 found to be important for FGF-2 induced-chemoresistance [39]. The mechanisms for these phenomena are still unclear, however, the development of specific S6K1 or S6K2 inhibitors will be helpful in exploring the specific functions of these two S6K isoforms.

It is reported that p70-S6K1 can be activated by mTORC1 and phosphoinositide-dependent kinase 1(PDK1) via the phosphorylation of Thr389 in the hydrophobic motif and Thr229 in the activation loop, respectively. Recent findings have shown that glycogen synthase kinase (GSK) also activates p70-S6K1 by phosphorylating Ser371 in the turn motif [40]. The major S6K substrates involved in translation regulation are ribosomal protein S6 (rpS6), eIF4B, eukaryotic elongation factor 2 (eEF2) kinase (eEF2K) and programmed cell death 4 protein (PDCD4) (Fig. 2).

rpS6, a key component of the small 40S ribosomal subunit, was known as the first identified substrate of S6K and five residues including Ser235, Ser236, Ser240, Ser244 and Ser247 in the C-terminus of rpS6 can be phosphorylated by S6Ks [36], the other two serine residues of rpS6, Ser235 and Ser236, can be phosphorylated by another 90-kDa ribosomal S6 kinase (RSK) [41]. Experiments using mice with wild-type rpS6 substituted by a phosphorylation negative mutant displayed severe growth defects which were also observed in S6K1/2 deficient mice [42]. Thus, it was proposed that the hyper-phosphorylation of rpS6 by S6Ks is implicated in the regulation of cell growth. However, the expression of phosphorylation negative rpS6 mutant showed a moderate up-regulation of global protein synthesis, whereas knockout of S6Ks had only a slight effect on overall translation rates [36, 42]. Therefore, the molecular mechanism for the influence of S6Ks and rpS6 on translation remains unclear.

eIF4B and eIF4H are two accessory factors that enhance the RNA-unwinding activity of eIF4A by promoting its processivity and switching its directionality. eIF4B can be phosphorylated by several members of the AGC kinase family on Ser406 (S6K and RSK) and Ser422 (S6K, RSK and AKT) in a context-dependent manner [43], following which activated eIF4B promotes the translation of mRNA and stimulates cell proliferation and survival.

Human eukaryotic elongation factor 2 (eEF2) kinase (eEF2K) functions as a negative regulatory factors of protein synthesis via phosphorylation and inhibition of eEF 2[44]. eEF2K is also a substrate of S6Ks, being phosphorylated and inactivated by S6Ks, as well as RSK and ERK1/2, resulting in increasing eEF2 function and elongation rates [45, 46]. PDCD4 is reported to be a pro-apoptotic factor and has been proposed to possess tumor suppressing properties. It is thought that PDCD4 interferes with the binding of eIF4G to eIF4A and blocks eIF4G and eIF4A interaction, leading to the inhibition of eIF4A helicase activity and the following suppression of cap-dependent translation [47]. Upon stimulation by growth factors, PDCD4 can be promptly phosphorylated on Ser67 and Ser457 by S6Ks and AKT, resulting in its degradation by the E3-ubiquitin ligase β-TrCP [48].

**Fig. 3:**
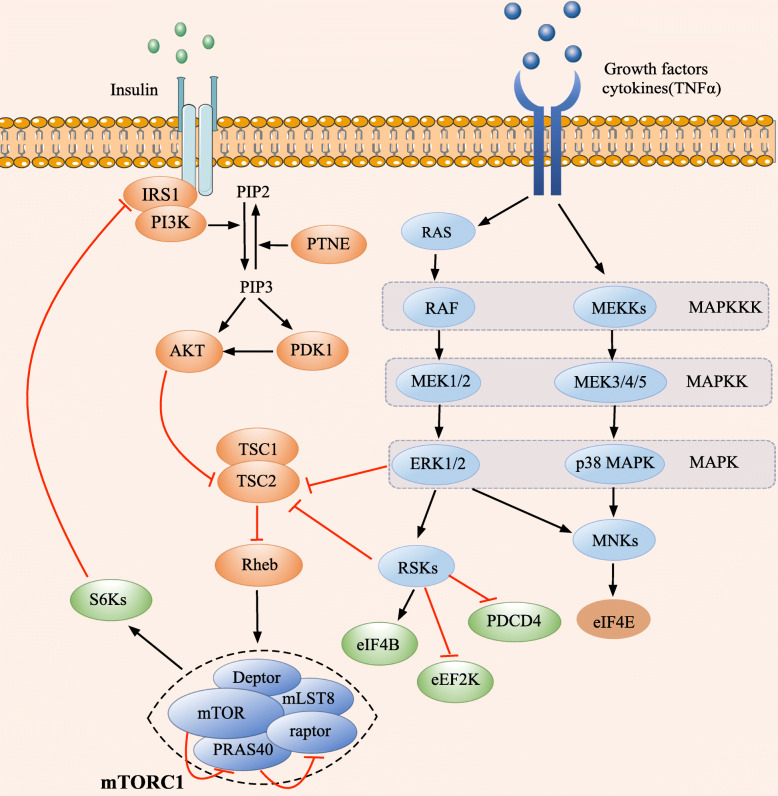
Schematic representation of PI3K and MAPK signaling to mTORC1. Insulin, growth factors and other stimuli activate mTORC1 signaling through binding and activating RTKs located at the membrane, following which PI3K/AKT and RAS-MAPK integrate these extracellular stimulating signals and convert them into intracellular signals. TSC consists of TSC2 and the scaffolding protein TSC1. The major target of AKT, ERK and RSK involved in the regulation of mRNA translation is TSC2, which is a GAP towards Rheb, and converts Rheb from its active GTP-bound form to the inactive GDP-bound form. Rheb is a small GTPase that stimulates the activation of mTORC1 in its GTP-bound active form. The phosphorylation of TSC2 by AKT, ERK and RSK impedes its GAP activity towards Rheb, resulting in increased Rheb-GTP levels and mTORC1 activation. The major targets of RAS-ERK and RAS-p38 MAPK are RSKs and MNKs. MNKs directly phosphorylate eIF4E on Ser209 which is thought to be the only post-translational modification of eIF4E, this phosphorylation of eIF4E enhances its ability to stimulate mRNA translation. In addition to TSC2, eIF4B, PDCD4 and eEF2K are also the major substrates of RSKs, as illustrated in Fig. 2 and in the text. Black arrows and red T-bars represent stimulatory and inhibitory signals, respectively

Additionally, S6Ks play a key role in triggering a negative regulatory feedback loop that restrains the activation of insulin-PI3K/AKT-mTORC1 pathway through the phosphorylation and inactivation of insulin receptor substrate 1 (IRS1), a major substrate of insulin receptor tyrosine kinase and crucial component in insulin signaling [49]. The limited therapeutic efficacy of rapamycin and rapamycin-induced AKT activation or persistent inhibition of S6K1 has been proposed to be caused by the loss of this negative regulatory feedback loop (Fig. 3).

## Major signaling pathways involved in the regulation of mTORC1 and eIFs

Growth factors and hormones are efficient stimulators for cancer cell growth and proliferation through activating several signaling pathways to increase protein synthesis. The major receptors, signal pathways and targets involved in this process are receptor tyrosine kinases (RTKs) and G protein-coupled receptors (GPCRs), PI3K/AKT and MAPK signal pathways, and the mTORC1 and eIFs targets, respectively [30] (Fig. 3). The growth of cancer cells and associated protein synthesis consume a large number of amino acids and energy, in the form of ATP, which have significant influence on the activity of mTORC1 and eIFs functions via the mediation of Rag GTPase, LKB1-AMPK (AMP-activated protein kinase), and Wnt-GSK3β (glycogen synthase kinase 3β) signal pathways [30].

### mTORC1 and eIFs regulated by the PI3K/AKT pathway

Growth factors, hormones including insulin and insulin-like growth factor (IGF), and other agonist ligands activate PI3K through binding and activating RTKs, then PI3K converts phosphatidylinositol (3,4)-bis-phosphate (PIP2) to phosphatidylinositol (3,4,5)-tris-phosphate (PIP3), which in turn contributes to the activation of phosphoinositide-dependent kinase-1 (PDK1) and AKT. PIP3 levels are reversed by PTEN, which is a tumor suppressor phosphatase and tensin homolog, negatively regulating the PI3K/AKT pathway [50]. Tuberous sclerosis complex (TSC), a well-known suppressor of mTOR activity, is comprised of TSC2 and the scaffolding protein TSC1 [51]. TSC2 functions as a GTPase activating protein (GAP) towards Rheb (Ras homologue enriched in brain) and converts Rheb from its active GTP-bound form to the inactive GDP-bound form [52] (Fig. 3). Rheb is a small GTPase that promotes the activation of mTORC1 in active GTP-bound Rheb form [53, 54]. Active AKT, ERK and RSK phosphorylate TSC2 at multiple residues including Ser939, Ser981 and Thr1462, which are thought to impede its GAP activity, thus resulting in elevated levels of GTP-bound Rheb and enhanced mTORC1 activation [51] (Fig. 3).

Additionally, PRAS40 is a binding partner and suppressor of mTORC1 activity, activated AKT directly phosphorylates PRAS40, leading to its dissociation from mTORC1 and preventing its suppression of mTORC1 signaling to S6Ks and 4E-BP 1[17]. PRAS40 is also a direct target of mTORC1, the phosphorylation of PRAS40 by mTORC1 assists in the removal of its inhibitory effect on the downstream signaling of mTORC 1[55]. Thus, it is suggested that mTORC1-induced phosphorylation of PRAS40 is a positive regulatory feedback mechanism for PI3K/AKT-mTORC1 signal pathway (Fig. 3).

Although it is well known that mTOR is often hyper-activated by mutations in its upstream signaling molecules, which include PI3K activating mutations and the loss-of-function mutations or gene copy deletion of the tumor suppressor PTEN [56, 57]. Direct evidence for mTORC1 functions involved in tumorigenesis comes from Tuberous Sclerosis, a multi-system genetic disease caused by defects or mutations of TSC1 or TSC2, consequently resulting in the hyper-activation of mTORC1, leading to widespread but benign tumor growth [58]. The lack of malignant transformation of Tuberous Sclerosis and the limited progression of these benign tumors may be due to mTORC1-S6K1-mediated negative feedback via phosphorylation of IRS-1 and inhibition of its activity, strongly down-regulating the signal transduction of most RTKs and PI3K/AKT pathways [59].

The significant role of PI3K in tumor development and progression makes it a promising therapeutic target [60, 61]. Great efforts have been made to develop PI3K inhibitors, some of which are currently undergoing clinical evaluation. Most agents targeting PI3K in the early phase clinical trials are ATP-competitive kinase inhibitors, including pan-PI3K inhibitors such as Buparlisib (BKM120) and dual PI3K/mTOR inhibitors such as BGT226. Currently, the most effective single agent PI3K pathway inhibitor is idelalisib (previously called CAL101 or GS1101) which has been approved by the FDA (U.S. Food and Drug Administration) for patients with chronic lymphocytic leukemia or non-Hodgkin lymphoma [62, 63].

### mTORC1 and eIFs regulated by MAPK pathways

The Ras-MAPK signal cascade is a critical pathway for cancer cell proliferation, migration and resistance to drug therapy. Driver mutations in *Ras* genes were the first specific genetic changes identified in human cancer and were found in up to ~30% of all human tumors [64–66]. The Ras-MAPK signal pathway consists of three consecutive kinases: MAPK kinase kinase (MAP3K), MAPK kinase (MAP2K) and MAPK. MAP3K is typically activated by small GTPases such as Ras, following which MAP2K is phosphorylated and activated by activated MAP3K, and the activation of MAP2K in turn phosphorylates and activates MAPK, ultimately phosphorylating and activating transcription factors in the nucleus or translation factors and thus resulting in protein synthesis [65, 67]. This cascade is usually initiated by various stimuli from outside the cell. MAP3K is composed of three members, A-Raf, B-Raf and C-Raf (Raf-1), whilst MAP2K is composed of MEK1 and MEK2, further downstream ERK/MAPK members are ERK1 and ERK2, which are the final effectors of MAPK pathway [67]. In mammals, MAPKs are grouped into four major families: the classical ERKs/MAPKs family, p38 MAPKs, C-Jun N-terminal kinases (JNKs) and Big MAPK-1 (BMK-1)/ERK5. Three of them, i.e., ERK, JNK and p38 have been widely studied and extensively characterized [64, 65].

The major MAPK pathways involved in the regulation of protein synthesis are Ras-ERK/MAPK and Ras-p38 MAPK pathways [68]. Both Ras-ERK/MAPK and Ras-p38 MAPK are stimulated and activated by a broad variety of stimuli such as growth factors, cytokines and a diverse set of environmental stresses. Although many factors activate both Ras-ERK/MAPK and Ras-p38 MAPK pathways, growth factors and stress stimuli typically activate the Ras-ERK/MAPK and Ras-p38 MAPK signaling, respectively [69]. Many of substrates of ERK/MAPK and p38 MAPK have been demonstrated to control gene expression, the two major substrates, RSKs and MNKs (MAPK-interacting kinases), play a critical and direct role in the regulation of translation initiation [70, 71] (Fig. 3).

#### RSKs

The vertebrate RSKs (90 kDa ribosomal S6 kinase) family is composed of four highly similar isoforms, RSK1, RSK2, RSK3 and RSK4, which are 73~80% identical. With the exception of RSK4, all RSKs have been demonstrated to be ubiquitously expressed in every developing and adult human tissues detected [72]. RSK1, RSK2 and RSK3 are usually present in the cytoplasm of quiescent cells, but are largely translocated to the nucleus upon outside stimulation and the activation of ERK1/2. The most striking feature of the RSK family is that its members have two non-overlapping and functional kinase domains, the C-terminal kinase domain (CTKD) and the N-terminal kinase domain (NTKD). The CTKD of RSKs contributes to the response to an upstream stimulating signals from ERK1/2, and then transmitting the activating signals to the NTKD with high efficiency and fidelity. It is the NTKD that phosphorylates the substrates of the RSKs [73]. The NTKD has the properties and functions of the AGC (protein kinase A, G and C) family kinases, explaining why RSKs, AKT and S6Ks have shared substrates [74].

The first evidence indicating that RSKs may take part in the regulation of mRNA translation came 30 years ago when it was recognized as an rpS6 kinase in *Xenopus laevis* oocytes. Subsequent studies demonstrated that activated RSKs associated with polysomes and enhanced the phosphorylation of several ribosome-associated proteins [75]. With the use of rapamycin, S6K1 and S6K2 were identified as the principal rpS6 kinases operating in somatic cells [76]. Studies using the cells from S6K1/2- deficient mice further verified these discoveries, but also indicated that there were low levels of rpS6 phosphorylation on Ser235 and Ser236, which was dependent on ERK1/2 activation [36]. The role of this specific regulation remains unclear, however, these results propose that RSK affords a mTOR-S6K independent but ERK1/2 dependent signal for regulating mRNA translation initiation.

Ras-ERK/MAPK signaling influences the PI3K/mTOR pathway at diverse levels to control mRNA translation. Additionally, as mentioned above, RSK directly regulates and phosphorylates multiple components of the translation initiation apparatus including rpS6, eIF4B and eEF2K, which are also the substrates of S6Ks. As members belonging to AGC protein kinase family, RSK and S6K phosphorylate eIF4B on the same residue, resulting in its enhanced interaction with eIF3 and increased translation rates. However, the phosphorylation of eIF4B by RSK and S6K is in a growth factor dependent manner, and the two phosphorylation sites show different phosphorylation rates [43].

The phosphorylation of eEF2K and PDCD4 by RSK causes the inhibition of eEF2K kinase activity and PDCD4 degradation, respectively. eEF2K and PDCD4 are two major negative regulators in mRNA translation, thus, the elimination of their negative effects on translation through phosphorylation by RSK greatly promotes protein synthesis [45, 77]. In addition, since GSK3β phosphorylates eIF2B and inhibits its functions in mRNA translation, RSK-mediated phosphorylation and inhibition of GSK3β promotes cancerous proliferation [78].

#### MNKs

MNKs have four molecular isoforms including MNK1a, MNK1b, MNK2a and MNK2b generated by alternative splice. MNK2a displays a higher basal kinase activity than other isoforms owing to its continuous association with ERK1/2 [79]. The interaction between the N-terminal regions of MNKs and the C-terminal domain of eIF4G recruits MNKs to eIF4E, finally resulting in the phosphorylation of eIF4E on Ser209, which is the only post-translational modification of eIF4E [80]. eIF4E phosphorylation was suggested to be an important event in tumorigenesis and tumor progression [81]. As far as transforming ability being considered, the non-phosphorylated eIF4E S209A mutant is less efficient than its wild-type counterpart, *in vitro* and *in vivo* [80]. Furthermore, constitutively activated MNK1 promotes tumor progression in a way similar to eIF4E, and the kinase-inactive MNK mutant suppresses the proliferation of cancer cells *in vivo*, thus, suggesting the critical role of MNK/eIF4E pathway in tumorigenesis [82].

### Regulation of mTORC1 by amino acids through Rag GTPase

Currently, it is known that amino acids, particularly the branched chain amino acids, are indispensable nutrients for cancer cell proliferation and are used by cancers in various biosynthetic pathways and as a source of energy [83]. A significant step in deciphering mTORC1 activation by amino acids was made with the identification of the Rag GTPases as mediators of amino acid signaling to mTORC1 [84–86]. In *Saccharomyces cerevisiae*, amino acids activate TORC1 through the Vam6/VPS39-Gtr1/Gtr2 axis. Vam6/VPS39 functions as a GEF (guanine nucleotide-exchange factor) for the Gtr1 GTPase, which is a component of the vacuolar membrane-associated TORC1-Ego1/2/3 complex and causes TORC1 activation [84, 87]. In mammals, the Rag GTPases have four isoforms including Rag A, Rag B, Rag C and Rag D. Rag A and B, and Rag C and D are functionally overlapping proteins and share 90% and 80% sequence identity, respectively. Rag A binds to Rag C or Rag D, Rag B also binds to Rag C or Rag D to form a stable and active heterodimeric complex [88]. Under amino acids sufficient conditions, an active Rag complex is composed of GTP-binding Rag A or Rag B and GDP-binding Rag C or Rag D. The Rag GTPases are unable to directly promote kinase activity of mTORC1, but are able to recruit and anchor mTORC1 to the cytoplasmic surface of lysosomes and consequently enhancing mTORC1 activation by Rheb [86].

**Fig. 4:**
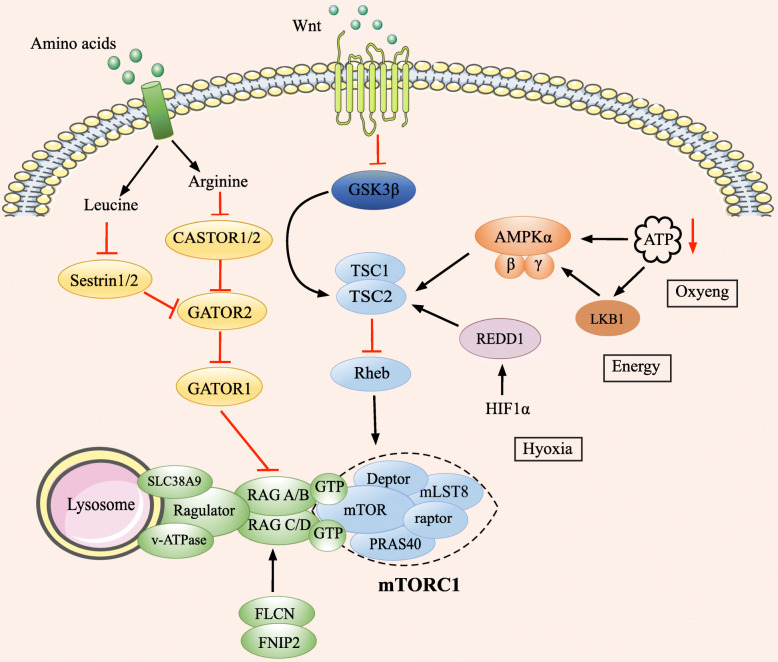
Schematic representation of amino acid, energy and Wnt signaling to mTORC1. Rag GTPases were identified as mediators of amino acid signaling to mTORC1. When an adequate supply of amino acids is present, an active Rag complex consists of GTP-bound Rag A or Rag B and GDP-bound Rag C or Rag D. The Rag complex is able to recruit and anchor mTORC1 to the lysosomal surface which facilitates mTORC1 activation by Rheb. Ragulator functions as a GEF for Rag A/B and also as a scaffold to help anchor the Rag complex to the lysosome. v-ATPase interacts with Ragulator and is required for mTORC1 activity. The GATOR1 complex functions upstream of the Rag complex as a GAP for Rag A/B GTPase and inhibits mTORC1 activity. The GATOR2 complex interacts with and inhibits GATOR1. Sestrin1/2 and CASTOR1/2 are cytosolic leucine and arginine sensor, respectively. The presence of leucine and arginine disrupts the association of Sestrin1/2 and CASTOR1/2 with GATOR2, resulting in the elimination of their inhibition towards GATOR2. SLC38A9 is an important lysosomal arginine sensor and amino acid transporter that directly interacts with Ragulator. FLCN and its binding partner FNIP2 were identified as Rag-interacting proteins with GAP activity for Rag C/D, but not for Rag A/B. Reduction in oxygen or energy levels are sensed by AMPK which can be activated by upstream kinase LKB1 under the conditions of the increased intracellular AMP/ATP and ADP/ATP ratios. The activated AMPK phosphorylates TSC2 and enhances its GAP activity towards Rheb-GTP, finally resulting in the inhibition of mTORC1 activity. Hypoxic stress also stabilizes the transcription factor HIF1α which drives the expression of REDD1. The latter is a negative regulator of mTORC1 activity. Additionally, the activated Wnt signaling pathway stimulates mTORC1 activity via GSK3β repression. Black arrows and red T-bars represent stimulatory and inhibitory signals, respectively

Ragulator functions as a GEF for Rag A/B and also as a scaffold to stabilize the Rag complex to the lysosome. v-ATPase associates with Ragulator and is required for mTORC1 activity [89]. GATOR1 (GAP activity towards Rags 1) complex acts as a GAP for Rag A/B GTPase and inhibits mTORC1 activity [90]. The GATOR2 complex interacts with and inhibits GATOR1 (Fig. 4).

Different amino acids have their specific intracellular sensors and activate mTORC1 via distinct pathways. Sestrin1/2 and CASTOR1/2 (cellular arginine sensor for mTORC1 complex1/2) are cytosolic leucine and arginine sensor, respectively [91–93]. Both leucine and arginine disrupts the association of sestrin1/2 and CASTOR1/2 with GATOR2, resulting in the elimination of their inhibition towards GATOR2 [94, 95] (Fig. 4). FLCN and its binding partner FNIP2 were identified as Rag-interacting proteins with GAP activity for Rag C/D, but not for Rag A/B [96]. SLC38A9 was recently characterized as a lysosomal arginine sensor and amino acid transporter that directly associates with Ragulator [97]. By regulating the expression levels of SLC38A,it was found that SLC38A plays a key role in mTORC1 activation by amino acids, especially arginine [98] (Fig. 4).

### Regulation of mTORC1 by energy levels and oxygen availability

Energy levels and oxygen availability fluctuate significantly during tumor progression and have great influence on cancer cell proliferation [99]. Alterations in cellular energy levels are detected by AMPK, which functions as the energy sensor for mTORC1 [100]. AMPK is a heterotrimeric complex that is composed of a catalytic subunit α and two regulatory subunits β/γ. AMPK can be stimulated and activated by various forms of intracellular stress, particularly the elevated cytosolic AMP/ATP or ADP/ATP ratios, because AMP or ADP binding to the regulatory AMPK γ subunit promotes its phosphorylation and activation by upstream kinase LKB1 [101]. The phosphorylation of AMPK activates TSC2 and promotes its GAP activity towards Rheb-GTP, finally leading to the suppression of mTORC1 activity [102, 103] (Fig. 4).

The phosphorylation sites of TSC2 for AMPK are different from those for Akt, and TSC2 phosphorylation by Akt is suggested to suppress its GAP activity and promotes mTORC1 activation. Additionally, other studies indicate that activated AMPK can inhibit mTORC1 in a TSC2-independent manner. These studies reported that two well-conserved serine residues in the mTOR interacting partner Raptor can be directly phosphorylated by AMPK. The phosphorylation of Raptor subsequently recruits 14-3-3 to bind to Raptor and leads to the suppression of mTORC1 activity [104]. Therefore, Akt-TSC2-mTORC1 and LKB1-AMPK or TSC2-mTORC1 play a critical role in balancing cell growth and energy supply.

Additionally, it is well known that the activation of the Wnt pathway inhibits GSK3β, resulting in the enhanced stability of transcription factors such as β-catenin, and the transcription of a wide range of growth-promoting genes. Wnt signaling also has a great effect on mTORC1 activity. The Wnt signaling and mTORC1 pathways are linked by GSK3β which also phosphorylates TSC2, and these phosphorylation events require previous phosphorylation of TSC2 by AMPK. The sequential phosphorylation of TSC2 by AMPK and GSK3β promotes the GAP activity of TSC2 towards Rheb-GTP, leading to the suppression of mTORC1. Therefore, the activated Wnt signaling pathway enhances the mTORC1 activity via GSK3β repression (Fig. 4).

In addition, oxygen availability also plays an important role in the activation of mTORC1. Reduction in oxygen limits ATP production, thereby resulting in the activation of LKB1 and AMPK, and the suppression of mTORC1. Hypoxic stress also stabilizes a transcription factor HIF1α (hypoxia-inducible factor 1α) which promotes the expression of a range of proteins such as REDD1 (regulated in development and DNA damage response) [105]. REDD1 impedes the activity of mTORC1 by competing with 14-3-3 for TSC2 binding to restrain the inhibitory binding of 14-3-3 to TSC2, finally resulting in the suppression of mTORC1 signaling [106] (Fig. 4).

## eIFs mis-regulation in human cancer and potential targets for cancer therapy

### eIF1 and eIF1A mis-regulation in human cancer

eIF1 and eIF1A are essential for the formation of PIC which binds to 5’-Cap region and shifts to the translation initiation codon. eIF1 was identified as a genotoxic and endoplasmic reticulum stress-inducible protein [107]. The induced expression of eIF1 is detected in various human cancer cell lines treated with UV or base damaging agents (Table 1) [107]. The mutations of eIF1A within its unstructured N-terminal tail are frequently observed in several types of malignancies. Recently, it was found that eIF1A differentially affects the translation of certain mRNAs. eIF1A knockdown causes a significant enrichment of cell cycle-related mRNAs, which are predominantly characterized by the long length of their 5’-UTR. Conversely, eIF1A knockout leads to an increased rate of 5’UTR translation initiation at a near cognate start codons, suggesting a predominant role of eIF1A in inhibiting 5’UTR translation. More importantly, cancer-associated mutants of the eIF1A N-terminal tail enhance the eIF1A functions towards a long 5’UTR and promote the expression of long 5’UTR-containing genes which control cell division cycle [108].

**Table 1:** Aberrant expression of eIFs in human cancer

eIFs	Dysregulation	Type of cancer
eIF1A/ eIF1AX	Mutation	Uveal melanomas, papillary thyroid carcinoma (PTC), anaplastic thyroid carcinomas (ATC), leptomeningeal melanocytic neoplasms (LMNs) [2, 108]
eIF2α	Overexpression	Non-Hodgkin’s lymphoma, melanocytic neoplasm, NSCLC, gastrointestinal cancer, and brain tumor [2, 109, 110]
eIF3a	Overexpression	Brain cancer, cervix cancer, lung cancer, stomach cancer and colorectal cancer [2, 10]
eIF3c	Overexpression	Meningioma and testicular seminoma [2]
eIF3e	Downregulation	Breast cancer, NSCLC and prostate cancer [2, 111]
eIF3f	Downregulation	Melanocytic neoplasm, pancreatic cancer, breast and ovary cancer [2, 112, 113]
eIF3h	Overexpression	Breast cancer, prostate cancer, hepatocellular carcinoma, NSCLC and colorectal cancer [2, 111, 114–119]
eIF4E	Overexpression/hyperphosphorylation	Breast cancer, lung cancer, prostate cancer, colorectal cancer, skin cancer, head and neck cancer and cervical cancer [2, 120–125]
eIF5A	Overexpression	Cervical cancer, NSCLC and colorectal cancer [2, 126]
eIF6	Overexpression	Colorectal cancer, head and neck carcinoma, malignant mesothelioma and NSCLC [2]

### eIF2 mis-regulation in human cancer

eIF2 is a heterotrimeric tRNA carrier composed of the components eIF2α, eIF2β and eIF2γ, which together take part in the formation of the eIF2-Met-tRNAi-GTP heterotrimer complex. During translation initiation, eIF2α-GTP is hydrolyzed to yield eIF2α-GDP. eIF2B is a GEF which promotes the recycling of GTP bound to eIF2α, and this process is blocked by the phosphorylation of eIF2α [127]. In other words, the GEF activity of eIF2B towards eIF2α can be suppressed by eIF2α phosphorylation.

Other studies have also showed that tumorigenesis and progression in mouse models are enhanced when the phosphorylation of eIF2α was inhibited through interfering with the expression of the eIF2α upstream kinase or by a phosphorylation negative eIF2α mutant [10, 128]. Therefore, the elevated eIF2α phosphorylation induced by some stress stimuli usually results in decreased proliferative capability of cancer cells through inhibiting global protein synthesis. Moreover, the increased expression level of eIF2α has been identified in the malignant lymphocytes from patients with non-Hodgkin’s lymphoma subtypes (Table 1) [109]. It was also found that eIF2α expression is significantly increased in both benign and malignant melanocytic tumors, where elevated levels of eIF2α may drive cancer initiation, but are insufficient to promote malignant progression [110]. Conversely, when neurocytoma is considered, the phosphorylation of eIF2α has not been found to be associated with benign or malignant brain cancers [129], and in some animal models, inactivating PKR (double-stranded RNA-dependent protein kinase), an upstream kinase of eIF2α through genetic mutation of its catalytic domain, has no effect on cancer progression [10]. Therefore, the functions of eIF2α and its phosphorylation in cancer remain unclear, and may depend on stage and grade of cancers. A hypothesis for this puzzle is that the level of eIF2α phosphorylation is increased in the early stages of cancers in order to respond to serious microenvironmental stresses to reduce protein synthesis and facilitates cancer cell survival [10].

### eIF2 as a potential target for cancer

**Fig. 5:**
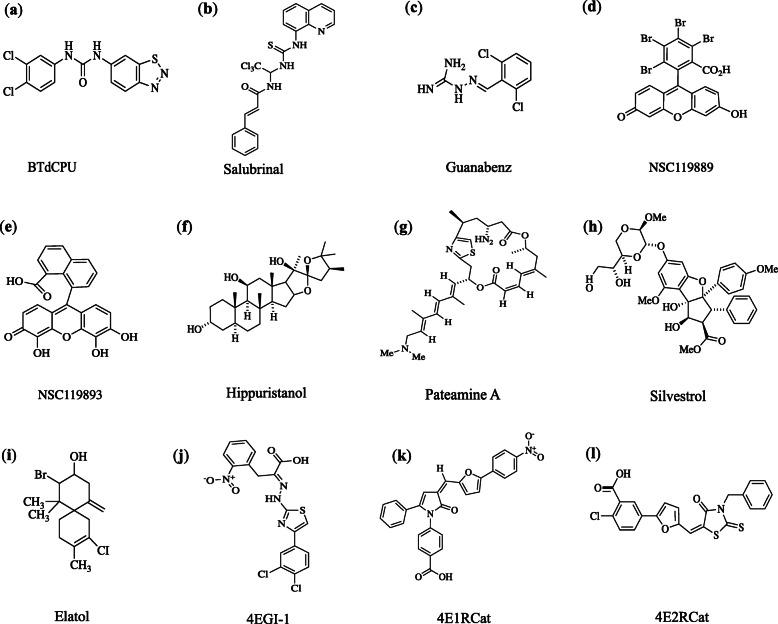
The direct inhibitors of translation apparatus. The structures of compounds are derived from PubChem. BTdCPU is an activator of HRI which can phosphorylate eIF2α. Salubrinal and guanabenz are inhibitors of phosphatase and inhibit eIF2α dephosphorylation. NSC119889 and NSC119893 are direct inhibitors of eIF2-GTP-Met-tRNAi^Met^ ternary complex and prevent the binding of tRNAi^Met^ to eIF2. Hippuristanol, pateamine A and silvestrol are inhibitors of eIF4A. Elatol is an eIF4A-specific inhibitor. 4EGI-1, 4E1RCat and 4E2RCat are inhibitors of eIF4E-eIF4G association

eIF2α can be phosphorylated by several kinases in response to distinct forms of stress, finally resulting in the global inhibition of protein expression, but paradoxically accompanied with the increased and selective translation of a subset of mRNAs encoding proteins that promote cellular adaptations. However, the sustained phosphorylation of eIF2α induced by PKR, PERK (PKR-like ER kinase), or HRI (heme regulated inhibitor kinase) causes cell apoptosis. Therefore, elevating the level of eIF2α phosphorylation would be a promising strategy to treat cancer. Using a cell base, BTdCPU and related *N*,*N*’-diarylureas were shown to activate HRI and phosphorylate eIF2α, displaying attractive antitumor effects *in vitro* and *in vivo* [130, 131] (Fig. 5a).

Another strategy to increase the phosphorylation of eIF2α is the use of phosphatase inhibitors, such as salubrinal and guanabenz (Fig. 5b and Fig. 5c). Salubrinal was identified as a selective phosphatase complexes inhibitor and inhibited ER (endoplasmic reticulum) stress-mediated apoptosis [132]. However, the sustained hyper-phosphorylation of eIF2α with high doses of salubrinal treatment resulted in cancer cells apoptosis [133]. Guanabenz, a selective agonist for alpha-2 adrenoceptor used as an antihypertensive drug, binds to a regulatory subunit of protein phosphatase 1, selectively inhibiting the stress induced-dephosphorylation of eIF2α [134]. Guanabenz was reported to increase the survival of Hela cells exposed to cytotoxic ER stress, while it attenuated the malignant phenotype and tumor growth in mouse models of breast cancer [135]. In addition, eIF2-GTP-Met-tRNAi^Met^ ternary complex is also a potential target for the inhibition of translation initiation, NSC119889 and NSC119893, brominated derivatives of fluorescein, have been found to directly target ternary complex formation and prevent the binding of tRNAi^Met^ to eIF2 (Fig. 5d and e) [136].

### eIF2B mis-regulation in human cancer

eIF2B, a GEF for eIF2, is considered to be the master regulator of translation initiation and composed of five subunits (α, β, γ, δ and ε) which are encoded by genes *eIF2B1*, *eIF2B2*, *eIF2B3*, *eIF2B4* and *eIF2B5*, respectively [137]. The mutations in the *eIF2B2* and *eIF2B5* genes have been reported to be the cause of an inherited disease called vanishing white matter (VWM) [138]. Later, it was found that mutant eIF2Bε protein results in the deficiency of astrocyte function, and further contributes to the loss of white matter in VWM leukodystrophy [139]. As the largest subunit, eIF2Bε contains the catalytic domain and promotes GDP/GTP exchange on eIF2. Additionally, eIF2Bε is found to be upregulated in live cancer and its expression is related to histologic grade, clinical stage and vital status. Furthermore, high eIF2Bε expression correlates with poor prognosis and is an independent risk factor for liver cancer, while the downregulation of eIF2Bε expression leads to reduction in GEF activity and global protein synthesis, as well as significant reduction in cell growth rate, colony formation and tumor progression in nude mice [140].

### eIF3 complex mis-regulation in human cancer

The eIF3 complex is the largest and most complex initiation factor and has 13 isoforms known as eIF3a to eIF3m, which are organized in various combinations to assemble the eIF3 complex. Of these eIF3a, eIF3b, eIF3c, eIF3e, eIF3f and eIF3h are the central subunits of eIF3 complex [141]. Its important functions in protein synthesis endows eIF3 complex with a prominent role in tumorigenesis. The overexpression of eIF3 complex subunits including eIF3a, eIF3b, eIF3c, eIF3h and eIF3i individually promotes the malignant transformation of fibroblast cells by stimulating global protein synthesis and the translation of specific mRNAs encoding growth regulators [142].

Both eIF3a and eIF3c have been found to be overexpressed in various cancers (Table 1) [10]. However, our previous studies have indicated that eIF3a inhibits C-Raf activation and that the down-regulated expression of eIF3a by small interfering RNA promotes ERK activation. Thus, these observations lead to the interesting hypothesis that mitogenic signaling may be regulated by free subunits of translation factors in order to make sure that the cellular response to mitogenic stimulation remains consistent with the cellular capability of protein synthesis [143].

Regarding eIF3h, it has been observed to be frequently amplified along with the proto-oncogene *Myc* in breast and prostate cancer. Additionally, the levels of eIF3h expression are positively associated with the poor differentiation and aggressive growth of prostate cancer [114]. Likewise, the increased expression of eIF3h was related to proliferation, migration and invasion of human hepatomas [115]. The magnification of the *eIF3h* gene has been identified in colorectal cancers and non-small cell lung cancer (NSCLC) through Genome-wide analyses and fluorescence in situ hybridization (FISH), respectively (Table 1) [111, 116–118]. More importantly, *eIF3h* and *Myc* co-amplification markedly improve the response and survival of NSCLC patients treated with epidermal growth factor receptor (EGFR) tyrosine kinase inhibitor such as gefitinib [119]. eIF3e locus was originally identified as the integration region of mouse mammary tumor virus (MMTV), resulting in the production of a truncated mutant version of eIF3e in mammary epithelial cells, alveolar epithelial cells and mouse embryonic fibroblast cell line, where it leads to the malignant transformation of cells [144]. The reduced expression of eIF3e has also been reported in 37% of patients with breast cancer and 31% of NSCLC patients (Table 1) [145]. On the contrary, the overexpression of wild-type full-length eIF3e do not promote malignant transformation, indicating a possible tumor suppressor role for wild type eIF3e and an oncogenic potential of truncated eIF3e.

eIF3f is the only central component of eIF3 complex which has been found to be down-regulated in melanoma and pancreatic cancer (Table 1). Enforced overexpression of eIF3f inhibits protein synthesis and cell proliferation, and induced apoptosis in melanoma and pancreatic cancer [112], whereas knocking down the expression of eIF3f protects melanoma cells from apoptosis, indicating eIF3f may function as a negative regulator for translation. Indeed, the reduced transcription levels of eIF3f mRNA are usually detected in tumors including breast cancer, pancreatic cancer, ovarian cancer and melanomas [113]. Therefore, these results suggest that eIF3e and eIF3f may function as negative regulators of mRNA translation.

### eIF4F complex mis-regulation in human cancer

It is universally acknowledged that eIF4F plays a key role in the Cap-dependent mRNA translation [2] and indeed the abnormal activity of eIF4F complex has been detected in many tumors, resulting in the selective expression of proteins involved in tumorigenesis and metastasis [7]. As mentioned above, the eIF4F complex is composed of eIF4E (a small Cap-binding protein), eIF4A (an ATP-dependent RNA helicase) and a large scaffolding protein eIF4G. eIF4G associates with eIF4A and eIF4E and provides a scaffold which is necessary for the formation and activity of eIF4F complex [24]. The functions of the eIF4F complex are strictly regulated by several of its interacting proteins, such as the eIF4A-binding protein eIF4B, eIF4H and PDCD4, and eIF4E-binding proteins 4E-BPs.

eIF4B and eIF4H activate eIF4A [2], whereas PDCD4 suppresses eIF4A activity [47]. 4E-BPs are inhibitory protein for eIF4E [26]. PABP is a binding protein for eIF4G. As discussed above, the functions of the eIF4F complex in mRNA translation are directly controlled by several signaling pathways which potentially lead to tumor development and progression. eIF4E, the core subunit of eIF4F complex, is frequently overexpressed in many human cancer and most closely correlated with tumorigenesis and metastasis.

eIF4E functions involved in translation are regulated by its availability or expression levels and its phosphorylation at Ser209. The availability of eIF4E and its phosphorylation are regulated by 4E-BPs and eIF4G-associated kinases MNK1 and MNK2, respectively. The 4E-BPs compete with eIF4G for its binding site on eIF4E, the binding of 4E-BPs to eIF4E is reversible and regulated by mTORC1-dependent phosphorylation of 4E-BPs, thus when phosphorylated by mTORC1, hyperphosphorylated 4E-BPs are incapable of binding eIF4E, enabling the association of eIF4E with eIF4G and the formation of eIF4F complex [30]. Elevated levels of phosphorylated 4E-BP1, which are suggestive of higher levels of eIF4F complex, were detected in advanced prostate cancer [146, 147]. Similarly, reduced 4E-BP1 expression and elevated levels of 4E-BP1 phosphorylation in some cancers were positively correlated with higher grade tumors and reduced survival [148, 149]. The phosphorylation of eIF4E is promoted by its interacting protein eIF4G, which recruits MNK1 and MNK2 to phosphorylate eIF4E [150].

Several compelling lines of evidence from animal studies indicate that the elevated levels of eIF4E phosphorylation are closely related to the development and progression of cancer [81, 151, 152]. Perhaps the most direct evidence to support the role of eIF4E phosphorylation in cancer was obtained from a series of eIF4E or MNK1/2 mutants which were used to test their contribution to the lymphoma tumorigenesis in the Eμ-Myc transgenic mouse lymphoma model [82]. Mice treated with cells stably expressing the eIF4E S209A mutant that eliminates eIF4E phosphorylation were defective at promoting tumorigenesis. By contrast, a reciprocal phosphomimetic serine to aspartic acid mutation induced accelerated tumor onset which is comparable to that of wild type eIF4E [82]. Furthermore, a dominant negative MNK1 mutant which is unable to phosphorylate eIF4E inhibited the *in vivo* proliferation of tumor cells promoted by mutations that lead to the deregulation of protein synthesis [82].

The phosphorylation of eIF4E did not significantly increase global protein synthesis but stimulated the expression of anti-apoptotic and pro-invasion proteins [82, 151, 153]. The inhibition of eIF4E has been shown to have therapeutic potential for the treatment of several types of cancer [154–158]. Additionally, the overexpression of eIF4E has been found in breast [117], lung [120], prostate [121], colorectal [122], skin [123], head and neck [124], and cervical cancers (Table 1) [125], and elevated expression levels of eIF4E are strongly related to poor prognosis and decreased survival.

### eIF4F complex as a potential target for cancer therapy

There are several strategies to develop drugs that impede the functions of eIF4F complex: (1) inhibiting eIF4A helicase activity, (2) blocking eIF4E binding to the m^7^GpppN cap structure, (3) uncoupling eIF4E- eIF4G association, (4) inhibiting eIF4E expression.

#### Inhibitors of eIF4A

One way to limit eIF4F complex-dependent translation initiation is to target eIF4A, the only known enzymatic component of the complex. There are three small-molecule compounds, hippuristanol, pateamine A and silvestrol, which have been extensively characterized as inhibitors of eIF4A, and are currently being developed as potential chemotherapies (Fig. 5f-h). Hippuristanol is an allosteric inhibitor of eIF4A, it allosterically inhibits the binding of both free eIF4A and bound eIF4A in the eIF4F complex to RNA, which in turn blocks eIF4A helicase and ATPase activities [159]. Pateamine A and silvestrol, paradoxically promote the RNA-binding ability, ATPase and helicase activities of eIF4A, while then the RNA-binding affinity of eIF4A is in a non-sequence-specific manner, leading to the removal of eIF4A from the eIF4F complex by RNA-mediated sequestration of eIF4A. Pateamine A is an irreversible translation inhibitor, probably duo to its covalent inhibition of eIF4A activity, and therefore shows strong toxicity *in vivo* [160, 161]. However, less toxic pateamine derivatives have been developed [162, 163]. In terms of preclinical efficacy, silvestrol has the highest potency *in vivo* and is the best-understood eIF4A inhibitor, mainly because of its better pharmacological tolerance and lower non-specific toxicity [164]. Elatol, a marine natural compound, is a novel eIF4A-specific inhibitor which functions through binding the target at key sites in the helicase core of eIF4A and shows broad activity against multiple tumor types (Fig. 5i) [165].

#### Inhibitors of eIF4E-cap interaction

eIF4E-mediated malignant transformation is in cap-dependent manner, because overexpression of eIF4E mutant with cap-binding defect shows no transforming and tumorigenic potential. Therefore the cap analogs are classical inhibitors of eIF4E-cap association. The original cap analogs are m^7^GDP, m^7^GTP or their derivates. Presently, about 80 analogs have been synthesized and have been exhaustively tested *in vitro* and *in vivo* [166]. However, these cap analogs usually show poor cell membrane permeability and instability *in vivo* [167]. To solve this dilemma, “pronucleotides” with attractive pharmacokinetic advantages have been synthesized. *N*-*7*-benzyl guanosine monophosphate tryptamine phosphoramidate pronucleotide, dubbed 4Ei-1, was shown to inhibit eIF4E-cap dependent translation and enhance chemosensitivity to gemcitabine. Another nucleoside analog, ribavirin, was also reported to be an eIF4E-cap inhibitor and an anti-eIF4E cancer therapeutic. Ribavirin is the first eIF4E inhibitors applied in clinical studies and showed benefits for patients with acute myeloid leukaemia [168] and has been approved by FDA (Food and Drug Administration) in the treatment of RSV (respiratory syncytial virus) and HCV (hepatitis C virus).

#### Inhibitor of eIF4E-eIF4G interaction

Another strategy to block eIF4E functions is to discover and design small molecule inhibitors that would interference with eIF4E-eIF4G interaction. Three inhibitors of eIF4E-eIF4G association, 4EGI-1, 4E1RCat and 4E2RCat, were identified by high-throughput screening of chemical libraries [169] (Fig. 5j-l). These molecules inhibited cap-dependent translation and exhibited activity against multiple cancer cell lines [169]. Recently, structural studies of eIF4E and 4EGI-1 complex showed that 4EGI-1 allosterically modify eIF4E by binding to its hydrophobic pocket which is distant from the eIF4G binding site. So eIF4E undergoes localized conformational changes, resulting in the suppression of eIF4E-eIF4G association [170].

#### Targeting eIF4E production with antisense oligonucleotides (ASOs)

Early researches using antisense oligonucleotides (ASOs)-based approaches to target eIF4E synthesis showed the feasibility of suppressing eIF4E expression to inhibit tumorigenesis, but accompanied with short half-life of these ASOs in vivo [171, 172]. Subsequently, novel ASOs with the second-generation back-bone antisense modifications were developed. These second-generation ASOs can overcome such drawbacks by incorporating multiple sugar-phosphate backbone modifications to improve nuclease resistance and promote stability in tissue. The results of second-generation ASOs targeting eIF4E are encouraging, these ASOs effectively suppressed eIF4E expression in tumors, and greatly attenuate tumor burden in breast and prostate xenograft models. Furthermore, no apparent toxicity was detected [172]. This was attributed to a relatively minimal impact on global protein synthesis (less than 20% reduction), especially the decrease in expression of proteins involved in proliferation, survival, metastasis and encoded by eIF4E-sensitive mRNA [172]. Additionally, mouse models with long-term inducible suppression of eIF4E indicated that eIF4E suppression is well bearable for many tissues and are completely reversible without any significant negative impact on the life and health of mice [173]. Taken together, these results indicate that targeting eIF4E production directly may be an attractive strategy toward effective therapy for the treatment of tumors.

### eIF5A mis-regulation in human cancer

eIF5A is an essential component of the translation initiation apparatus, acting as a GAP for eIF2-GTP, and has also been newly assigned functions in elongation [4]. eIF5A has two isoforms, eIF5A1 and eIF5A2, which are expressed from distinct but related genes. Of note, the amino acid Hypusine is found in eIF5A, presumably due to a special eIF5A post-translation modification pattern. It is essential for most known eIF5A activities and is recognized as a novel potential therapeutic target in the treatment of BCR-BAL-positive leukemias [174].

eIF5A is clearly a factor required for stimulating protein synthesis. Rapid depletion of eIF5A *in vivo* promptly lead to a 2-fold decrease in protein synthesis in *Saccharomyces cerevisiae*, whereas treatment a eIF5A-depleted lysate with purified eIF5A stimulated an approximate 2-fold increase in protein synthesis, which is dependent on new initiation, suggesting that eIF5A functions at or near the initiation step to stimulate the formation of the first peptide bond in protein synthesis [126].

Furthermore, eIF5A was showed to activate the peptidyl transferase of ribosomes, especially for the synthesis of polyproline by alleviating ribosomal stalling on polyproline sequences [175]. Recently, it was found that knockdown of eIF5A lead to global translation elongation and termination defects and also that eIF5A was able to alleviate stalling on many motifs besides polyproline tracts [176].

### eIF6 complex mis-regulation in human cancer

eIF6 is a rate-limiting factor for mRNA translation initiation, cell growth and transformation [177]. It functions as a ribosomal anti-association factor in translation initiation through blocking the interaction between the 40S and 60S ribosomal subunits, namely, impeding the formation of 80S subunits without mRNA. However, nucleolar eIF6 and cytoplasmic eIF6 are required for the biogenesis of 60S subunits and growth factor-stimulated translation, respectively [178]. Furthermore, an increasing number of studies have demonstrated that there is a highly aberrant expression of eIF6 in various types of human cancer including colorectal cancer, head and neck carcinoma, malignant mesothelioma, acute promyelocytic leukemia and NSCLC (Table 1) [2, 179]. Therefore, modulation of eIF6 activity or expression may exert an innovative treatment for cancers. In a murine model of lymphomagenesis, the impairment of cytoplasmic eIF6 activity leads to the inhibition of lymphomagenesis and tumor progression as well as a striking increase of survival without adverse effects [180, 181].

## Conclusions

Over the last two decades, notable progress has been achieved towards understanding how oncogenic signaling pathways, including Ras-MAPK and PI3K-Akt, regulate the components of translation initiation apparatus and subsequent mRNA translation in eukaryotic cells. This is particularly important, as the translation initiation factors are often primary targets of these signaling pathways, which include several oncogenes and are mis-regulated in cancers, making abnormal translation an universal characteristic of cancer cells with different genetic make-up. Almost all these signaling pathways employ mTOR to regulate the functions of eIFs and mTOR is recognized as the master regulator of protein synthesis. The changes in the expression of certain translation initiation factors (mostly increased expression) are related to the development and progress of specific cancers. In this context, multiple components of several oncogenic signaling pathways and translation initiation factors involved in mRNA translation have been identified as biomarkers with potential diagnostic, therapeutic and prognostic utility. Therefore, anti-tumor agents targeting the core components of protein synthesis and related signaling pathways represent novel promising anticancer drugs and can get over intra-tumor heterogeneity. The initial successes of therapeutics that target dysregulation of mRNA translation in cancer suggest promising potential in their transition from the bench to the bedside in the near future.

## Data Availability

Not applicable’ for this section.

## Abbreviations

4E-BP1: 4E-binding protein 1
5’UTR: 5’ untranslated region
AMPK: AMP-activated protein kinase
ASOs: Antisense oligonucleotides
CASTOR1/2: Cellular arginine sensor for mTORC1 complex1/2
CDK1: Cyclin Dependent Kinase 1
Deptor: DEP domain-containing mTOR-interacting protein
eEF2K: eukaryotic elongation factor 2 kinase
EGFR: Epidermal growth factor receptor
eIFs: eukaryotic initiation factors
FISH: Fluorescence in situ hybridization
GAP: GTPase activating protein
GATOR1: GAP activity towards Rags 1
GβL: GTPase β-subunit like protein
GEF: Guanine nucleotide-exchange factor
GPCRs: G protein-coupled receptors
GSK: Glycogen synthase kinase
HIF1α: Hypoxia-inducible factor 1α
HCV: Hepatitis C virus
HRI: Heme regulated inhibitor kinase
IGF: Insulin-like growth factor
IRS1: Insulin receptor substrate 1
JNKs: C-Jun N-terminal kinases
MAP 2 K: MAPK kinase
MAP3K: MAPK kinase kinase
MAPK: Mitogen-activated protein kinase
mLST8: mammalian lethal with Sec13 protein 8
MMTV: Mouse mammary tumor virus
MNKs: MAPK-interacting kinases
mRNA: messenger RNA
NSCLC: Non-small cell lung cancer
mTOR: mammalian target of rapamycin
mTORC1: mTOR complex 1
mTORC2: mTOR complex 2
PABP: Poly (A)-binding protein
PDK1: Phosphoinositide-dependent kinase 1
PDCD4: Programmed cell death 4 protein
PERK: PKR-like ER kinase
PKC: Protein kinase C
PI3K: Phosphatidylinositol 3-kinase
PIP2: Phosphatidylinositol (3,4)-bis-phosphate
PIC: Pre-initiation complex
PIP3: Phosphatidylinositol (3,4,5)-tris-phosphate
REDD1: Regulated in development and DNA damage response
Raptor: Regulatory protein activated with mTOR
Rheb: Ras homologue enriched in brain
Rictor: Rapamycin insensitive companion of mTOR
rpS6: ribosomal protein S6
RSK: Ribosomal S6 kinase
RSV: Respiratory syncytial virus
RTKs: Receptor tyrosine kinases
SGK: Serum glucose kinase
TOP: Terminal oligopyrimidine motif
TSC: Tuberous sclerosis complex
VEGF: Vascular endothelial growth factor
